# Effects of parametric feature maps on the reproducibility of radiomics from different fields of view in cardiac magnetic resonance cine images– a clinical and experimental study setting

**DOI:** 10.1007/s10554-025-03404-y

**Published:** 2025-04-23

**Authors:** Laura Jacqueline Jensen, Damon Kim, Thomas Elgeti, Ingo Günter Steffen, Lars-Arne Schaafs, Anja Cretnik, Bernd Hamm, Sebastian Niko Nagel

**Affiliations:** 1 Department of Radiology, Charité– Universitätsmedizin Berlin, Corporate Member of Freie Universität Berlin, Humboldt-Universität zu Berlin and Berlin Institute of Health, Hindenburgdamm 30, 12203 Berlin, Germany; 2 Department of Cardiology, Angiology and Intensive Care Medicine, Charité– Universitätsmedizin Berlin, Corporate Member of Freie Universität Berlin, Humboldt-Universität zu Berlin and Berlin Institute of Health, Hindenburgdamm 30, 12203 Berlin, Germany; 3 Department of Diagnostic and Interventional Radiology and Paediatric Radiology, Bielefeld University Medical School and University Medical Center East Westphalia-Lippe Protestant Hospital of the Bethel Foundation Academic, Burgsteig 13, 33617 Bielefeld, Germany

**Keywords:** Cardiac magnetic resonance imaging, Cine MRI, Radiomics, Reproducibility, Feature map

## Abstract

In cardiac MRI, the field of view (FOV) is adapted to the individual patient’s size, influencing spatial resolution and myocardial radiomics. This study aimed to investigate the effects of parametric feature maps on radiomics derived from cine images acquired with different FOV sizes on individuals without myocardial pathologies. In the clinical setting, cardiac MRI scans from clinical care were screened retrospectively for patients without pathological findings, neither in the MRI nor the medical history or follow-up, resulting in 61 included patients. In the experimental setting, 12 healthy volunteers were prospectively examined on a 1.5 Tesla MRI scanner with cine images acquired with three different FOVs (256 × 329 mm, 279 × 359 mm, 302 × 390 mm). One midventricular end-diastolic short-axis slice of the non-enhanced cine images was extracted for healthy volunteers and patients. The left ventricular myocardium was encompassed with regions of interest (ROIs). Ninety-three features were extracted using PyRadiomics. Images were converted to parametric radiomic feature maps using pretested software. ROIs were copied to the maps to retrieve the feature quantity. The variability of features across the different FOVs from the original images and feature maps was assessed with coefficients of variation (COVs) and rated stable at up to 10%. When derived from the original images, out of the 93 extracted features, only 24 (patients) and 29 (volunteers) revealed COVs < 10%. When extracted from the parametric maps, the number of stable features increased by 63% and 66%, with 39 (patients) and 48 (volunteers) features showing COVs < 10%, respectively. Software-computed parametric feature maps improve the reproducibility of radiomics across different FOVs in cardiac cine images of individuals without myocardial pathologies. Prospective investigations with different FOVs of a patient collective with myocardial pathologies could enhance the generalizability of the findings.

**Supplementary Information:**

The online version contains supplementary material available at 10.1007/s10554-025-03404-y.

## Introduction

Cardiac magnetic resonance (CMR) imaging has become indispensable in cardiovascular diagnosis [1, 2]. It has proven valuable in the diagnostic workup of inflammatory and ischemic heart disease, various cardiomyopathies, and rare diseases like amyloidosis or congenital heart disease [2]. Steady-state free precession (SSFP) cine sequences are routinely acquired to assess left and right ventricular function and anatomical abnormalities. Further sequences like late gadolinium enhancement (LGE) images and cardiac relaxometry allow myocardial tissue characterization [3, 4]. CMR analysis requires expertise, and subtle myocardial changes can be difficult or impossible to detect, particularly in non-contrast-enhanced images [4].

Different studies attempted to capture such subtle, subvisual changes in the myocardium by applying the radiomics approach [5–9]. This technique quantitatively extracts multiple features from medical images via high-throughput calculations based on the pixel gray level distribution [10, 11]. For example, Fan et al. could separate the myocardial area at risk in reperfused acute myocardial infarction into the final infarct and remote zone by analyzing the texture of T2 mapping images [12]. Although radiomics as imaging biomarkers or part of widely applicable trained models seem intriguing, they have not yet arrived in clinical care since their lack of robustness and reproducibility is one major issue [4, 13, 14]. Potential influencing factors were identified, including scanners, vendors, field strengths, sequence parameters, and volume of interest (VOI) size [4, 15–18].

In CMR, each patient’s field of view (FOV) is adjusted individually, influencing the spatial resolution of the resulting images [19]. Radiomics analysis of cardiac cine images has become particularly important due to a study by Vande Berg et al. in which cine images were analyzed to distinguish between patients with acute myocardial infarction and healthy subjects [20]. They report only one FOV size as a specific acquisition parameter for all 148 study subjects. Fan et al. omit information on the used FOVs in their radiomics study [12]. Particularly in qualitative MR sequences such as T1-, T2-weighted, and cine, radiomics are highly sensitive to differences in spatial resolution [21]. The influence of varying FOV size and spatial resolution on radiomics derived from cardiac MRI is disregarded in most studies. Systematic investigations on the reproducibility of myocardial features across varying FOV sizes possess a current gap in the field of radiomics research. Recently, parametric feature maps were presented as a tool to improve the reproducibility of radiomics across different volume of interest (VOI) sizes compared to conventional extraction [22–24]. In contrast to traditional radiomics approaches, in which features are extracted from a VOI in the original images, the parametric map technique translates the entire image stack to a map for each feature [24]. With this automated, software-based method, the original image is disassembled into voxels of definable size. For each voxel, the feature quantity is calculated and stored in the parametric feature map in the exact position as in the original image. The resulting parametric map represents the feature quantity in brightness. The quantity is also directly measurable with a VOI in a standard image viewer [24]. A superior role of parametric feature maps compared to conventional radiomic feature extraction was reported for liver and lung MRI, yet they have not been scrutinized for CMR images [23, 25]. Since there is a demand to improve the reproducibility of myocardial radiomics, applying parametric maps to cardiac MRI might serve as an advancement [21, 26]. As a novel approach, we aimed to investigate if parametric feature maps might also improve the reproducibility of radiomics across different FOV sizes to address the gap within the existing research. Therefore, this study intended to compare the reproducibility of radiomics derived from parametric feature maps to radiomics extracted conventionally in (1) retrospectively collected clinical data with inherently different FOV sizes between patients and (2) prospective, experimental examinations of healthy individuals with different FOVs.

## Methods

### Clinical setting

#### Study subjects

**Fig. 1:**
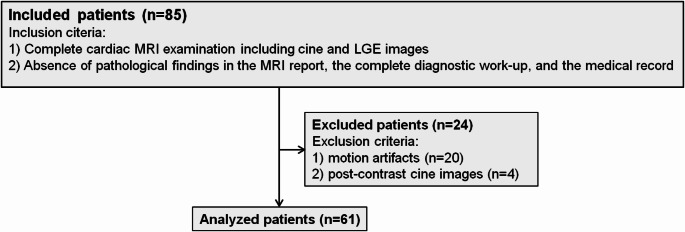
Flow chart of the included patients. The flow chart of the included patients is shown. Twenty patients were excluded due to motion artifacts, and four patients because cine images were acquired following the application of gadolinium (and this study aimed to examine non-contrast cine images)

This retrospective single-center study part was approved by the institutional review board (protocol code [EA1/104/19] and date of approval [5-14-2019]). All cardiac MRI examinations acquired on one MRI scanner between November 2015 and April 2017 were reviewed. Inclusion criteria were (1) a complete diagnostic cardiac MRI examination including CINE and late gadolinium-enhanced (LGE) images and (2) the absence of any pathological findings in the MRI report and the absence of cardiovascular pathologies in the complete diagnostic workup, and the medical history. Exclusion criteria were (1) motion artifacts and (2) CINE images acquired after administration of contrast media. Post-contrast cine images were excluded since different time points of image acquisition after contrast administration entail differences in contrast and because of the widespread clinical use of non-contrast cine imaging. A flow diagram of the included patients is shown in Fig. 1. Table 1 summarizes the demographic and clinical data of the analyzed patients. Detailed information on the indication for the MRI, comorbidities, and medication of the included patients can be found in the supplementary materials (S1).

**Table 1:** Details of the analyzed patients

Number of patients	61
Gender	25 female / 36 male
Median age (y)	26 (IQR: 20–30)
Median height (cm)	178 (IQR: 170–184)
Median weight (kg)	72 (IQR: 61–86)
Serum troponine	> 3 ng/l: 18 patients Median 8.5 ng/l (IQR: 4.25-29 ng/l)
	< 3 ng/l: 17 patients
	Not analyzed: 26 patients

IQR: interquartile range.

#### MRI examination

All examinations were performed in clinical routine care on one 1.5 Tesla MR system (Magnetom Aera, Siemens Healthineers, Erlangen, Germany) using the same imaging protocol with the FOV adjusted to the individual patient’s size to prevent aliasing [27]. Following localizer sequences, non-contrast-enhanced long-axis cine images (two/ three/ four-chamber) and short-axis cine images covering the entire left ventricle were acquired using a retrospective electrocardiogram-gated 2D steady-state free precession (SSFP) pulse sequence. Ten minutes after administration of 0.15 mmol/kg gadobutrol (Gadovist, Bayer AG, Leverkusen, Germany), LGE sequences in long-axis and short-axis views covering the entire LV were acquired using an inversion-recovery-prepared T1-weighted gradient echo sequence with manually adjusted inversion time based on the TI scout and a phase-sensitive inversion-recovery (PSIR)-based reconstruction. For the short-axis cine images, only one FOV per patient was used. Individual adjustment of the left-ventricular angulation and the field of view were the only settings that varied in the short-axis cine images. Standard scanning parameters of the short-axis SSFP sequences analyzed in our study are summarized in Table 2.

**Table 2:** Standard MR scanning parameters

Slice thickness/gap	8/2
TR/TE (ms)	46/1.3
Flip angle (deg)	69
Acquired matrix (pixels^2^)	192 × 125
Temporal resolution (ms)	35–44
Breathing regimen	multi-breath-hold
Patient position	Supine, head first (18-channel body phased-array coil positioned on chest)

TE: echo time. TR: repetition time

#### Image Preparation and segmentation

**Fig. 2:**
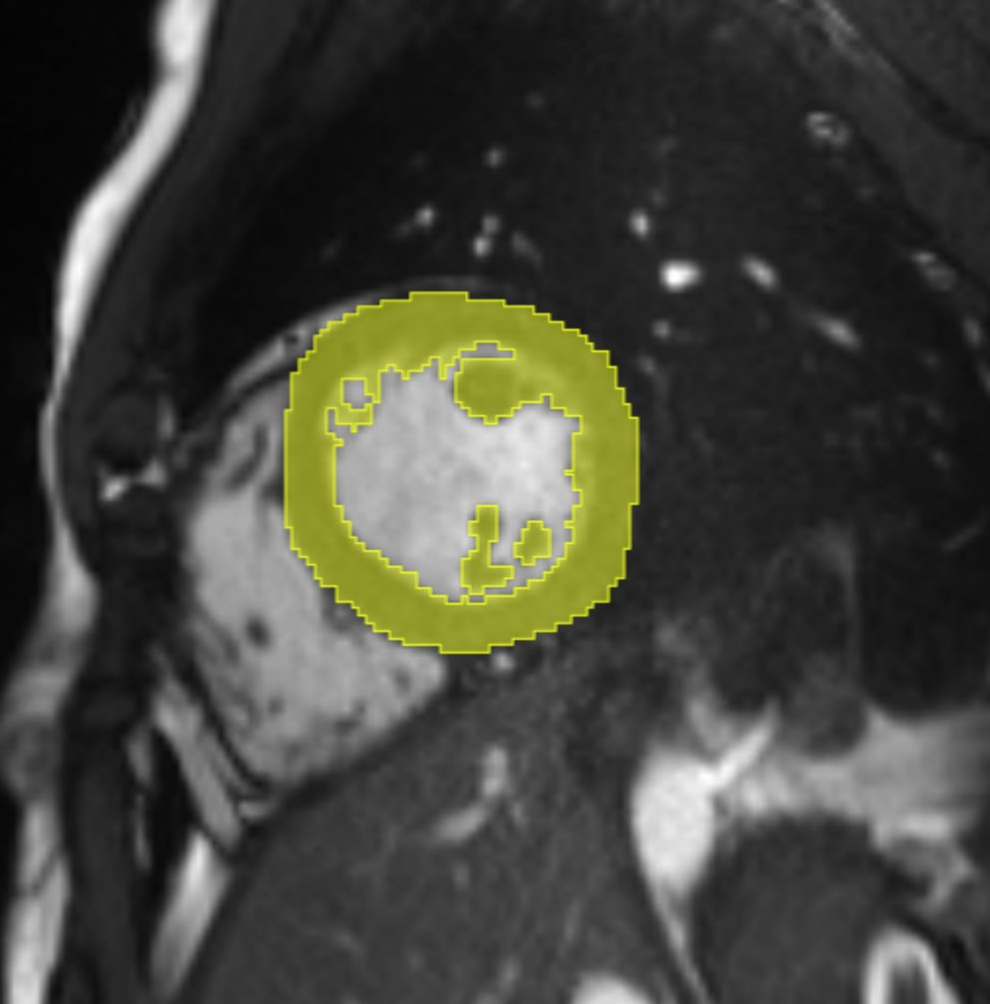
Semi-automated segmentation. Segmentation was performed semi-automated to generate reproductive regions of interest (ROIs) encompassing the left ventricular (LV) myocardium. First, the LV was marked manually with a ROI. Then, a threshold was applied, separating the myocardium from the blood pool

Since the cardiac cycle influences radiomic features from cardiac cine images, all images included in our study were end-diastolic frames [28, 29]. For each patient, a midventricular end-diastolic short-axis cine image slice was retrieved from the picture archiving and communication system (PACS) in the DICOM format. The slice was selected by descending the short cardiac axis from basal towards midventricular until the first slice on which the papillary muscles were distinctly visible. To enable further processing to parametric feature maps, the oblique images were rotated to the axial plane using 3D slicer (3D Slicer, Version 5.2.2, www.slicer.org), applying the “ignore orientation” option and saved in the NRRD-file format. To generate reproductive regions of interest (ROIs), we applied a standardized technique of marking the entire LV myocardium using 3D slicer (segmentations module): ROIs were drawn around the LV myocardium by marking the entire left ventricle and applying the threshold function (range 0-170). Segmentation was performed by a board-certified radiologist with over six years of experience. Since we used a standardized and semi-automated method for segmentation, inter-rater or intra-rater variability was not assessed. Figure 2 shows an exemplary segmentation.

#### Radiomics: conventional extraction from the original images

Using the open source-package PyRadiomics (Version 3.0) [30], 93 features were extracted: 18 first-order features (energy, total energy, entropy, kurtosis, maximum, minimum, mean, median, interquartile range (IQR), skewness, range, mean absolute deviation (MAD), robust mean absolute deviation (RMAD), root mean squared (RMS), variance, uniformity, 10th percentile, and 90th percentile) and 75 s- and second-order features (24 gray level co-occurrence matrix (GLCM) features, 14 gray level dependence matrix (GLDM) features, 16 gray level run-length matrix (GLRLM) features, 16 gray level size zone matrix (GLSZM) features, and 5 neighboring gray tone difference matrix (NGTDM) features [30]. All features available by PyRadiomics except shape features were calculated. Shape features were not considered since they are descriptors of the ROI shape, which is not expected to be altered by different FOV sizes. Settings of the feature extraction are provided in the supplementary materials (S2).

#### Radiomics: parametric feature maps

**Fig. 3:**
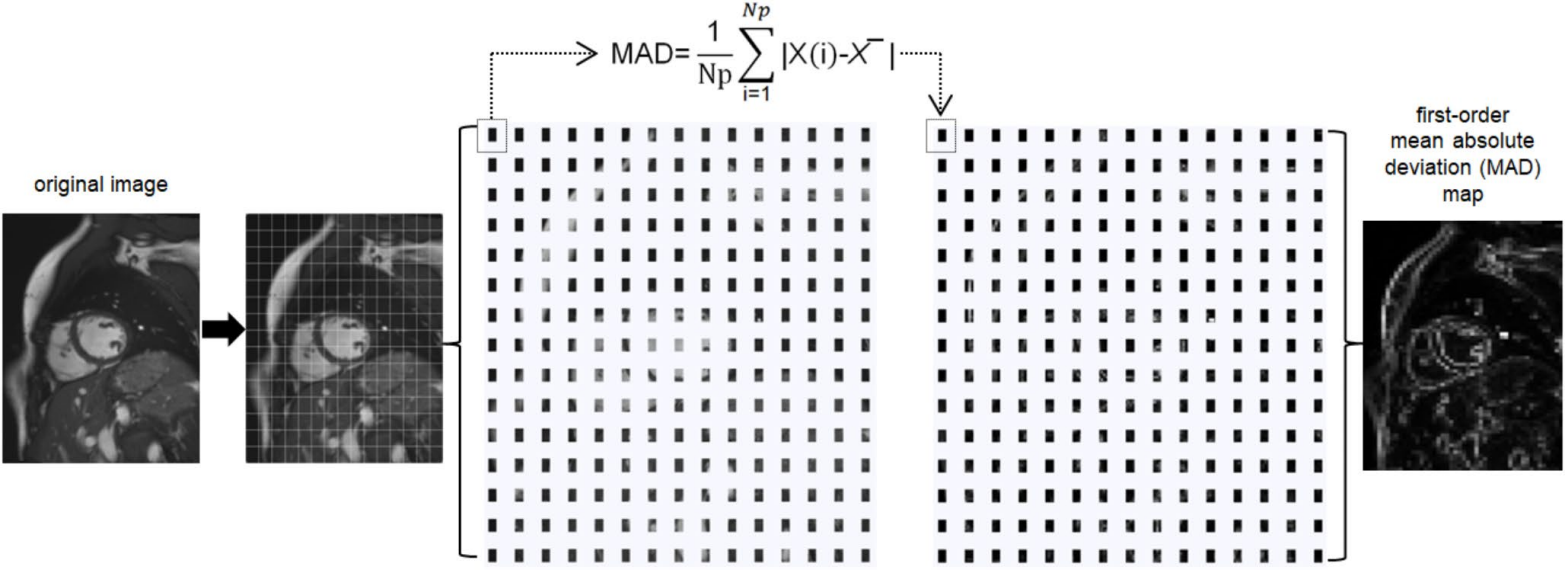
Workflow of the software pipeline The steps of the software script are shown schematically. First, the original image is dissembled into a grid of voxels (much finer as illustrated), the size of which can be adjusted in the script. Then, the feature quantity is calculated for each voxel and stored in the map with the same spatial information as in the original image. An exemplary map of mean absolute deviation is shown (the equations can be found on the PyRadiomics developers’ website: https://pyradiomics.readthedocs.io/en/latest/features.html). In the workflow of the software tool, maps for each feature available by PyRadiomics are calculated automatically for the input image

**Fig. 4:**
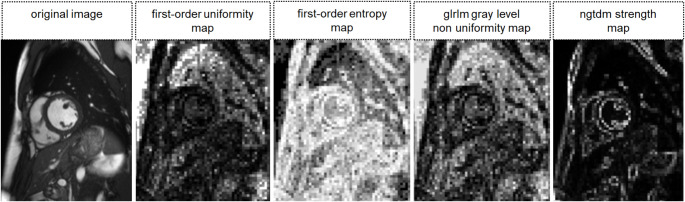
Exemplary parametric feature maps. Exemplary maps of first-order uniformity, first-order entropy, GLRLM gray level non uniformity, and NGTDM strength are shown. The higher the image quantity, the brighter the voxel in the resulting map. Numerical feature quantity can be retrieved directly from any location by extracting the mean

For every image, 93 parametric maps were computed using a pretested software tool [22, 24]. It dissembles the original image into small voxels of an adjustable size. The feature quantity is calculated for each voxel, representing the quantity with brightness in the exact location as in the original image, schematically shown in Fig. 3. Feature quantity can then be directly retrieved from any area on the map by extracting the mean (similar to using quantitative apparent diffusion coefficient (ADC) values in a standard viewer in clinical imaging). Following initial tests on optimizing computing time and spatial resolution of the maps, voxel size in the software script was set to x = 3 mm, y = 3 mm, and z = 8 mm (matching the slice thickness of 8 mm). In the workflow of the software tool, maps for each feature available by PyRadiomics are calculated automatically for the input image [30]. The software script used in this study is provided in the supplementary materials (S3). Figure 4 shows examples of calculated feature maps. ROIs were copied to the maps, and the quantity of each feature was retrieved by extracting the mean.

### Statistical analysis

Statistical analysis was performed in R (version 4.2.1, R Foundation for Statistical Computing) [31]. Coefficients of variation (COVs), i.e., the standard deviation normalized to the mean, were calculated to assess the variability [32]. For each feature, COVs were calculated across all included patients for the extraction (1) from the original images and (2) from the parametric feature maps. COVs up to 10% were rated stable and above 10% unstable, according to Mackin et al. [32, 33].

### Experimental setting

#### Study subjects

Twelve healthy subjects volunteered and were examined prospectively in March 2023 and February 2025. Details on the healthy subjects are compiled in Table 3.

**Table 3:** Details of healthy volunteers

	Subjects
Gender (f/m)	6/6
Age (y) median (IQR)	29 (28–34)
Height (cm) median (IQR)	174 (165–183)
Weight (kg) median (IQR)	67 (65–80)

#### MRI examination

**Fig. 5:**
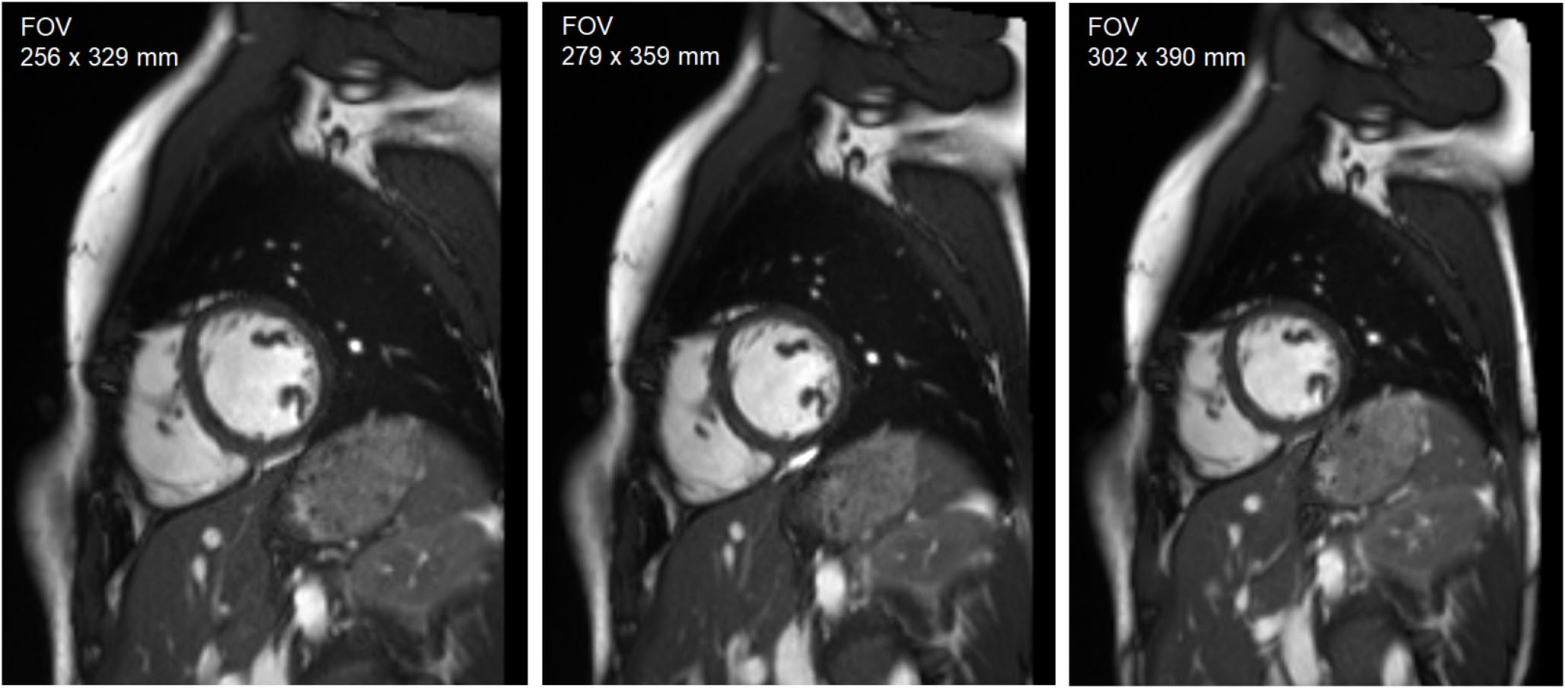
Short-axis cine images with different fields of view. Images of the study subjects were acquired with three different fields of view (FOV) (256 × 329 mm, 279 × 359 mm, 302 × 390 mm), otherwise maintaining the standard settings. Short-axis cine images of one subject for the three FOVs are shown. One midventricular image per FOV on the same level (slice level was correlated to the long-axis views to maintain the position) was used for analysis. The aim was to simulate different FOVs in routine clinical imaging

All Examinations were performed on one 1.5 Tesla MR system (Magnetom Aera, Siemens Healthineers, Erlangen, Germany). Following localizer sequences, non-contrast-enhanced long-axis cine images (two/ three/ four-chamber) and short-axis cine images covering the entire left ventricle were acquired using a retrospective electrocardiogram-gated 2D SSFP pulse sequence from clinical routine imaging. While maintaining the other standard settings, short-axis cine images were obtained thrice with different FOVs (256 × 329 mm, 279 × 359 mm, and 302 × 390 mm). Since FOVs are arbitrary in clinical routine imaging, the FOV variations were chosen to mimic real-world variability. According to the clinical study setting, the FOV and the left-ventricular angulation were the only parameters adjusted. The left ventricular angulation was kept unchanged for all three FOVs in each subject. Details of the short-axis SSFP sequence are listed in Table 4. Figure 5 shows images of the three different FOVs of one subject.

**Table 4:** Settings of short-axis 2D steady-state free precession pulse sequence

FOV (mm)*	256 × 329	279 × 359	302 × 390
Slice thickness (mm)	8		
TR/TE (ms)	36.63/1.38	35.2/1.32	34.1/1.28
Flip angle (deg)	58		
In-plane phase-encoding direction	anterior to posterior		
Acquisition matrix (pixels^2^)	224 × 157		
Breathing regimen	multi breath-hold		
Scanning position	supine, head first (18-channel body phased-array coil positioned on chest)		

FOV: field of view. TE: echo time. TR: repetition time. *subjects were scanned with three different FOVs

#### Image preparation, segmentation and radiomics

For each FOV, a midventricular end-diastolic short-axis cine image slice of the same level (defined by the localizer) for every subject was retrieved from the PACS in the DICOM format. Image preparation, segmentation, conventional feature extraction, and computation of parametric maps followed by feature extraction were performed as described in the clinical study part. For each file (36 files: three different FOVs for each of the 12 subjects), 93 parametric maps were computed and analyzed.

### Statistical analysis

COVs were calculated with R and rated as in the clinical study setting. COVs were calculated separately for each subject to assess variability across the three different FOVs.

## Results

### Clinical setting

**Fig. 6:**
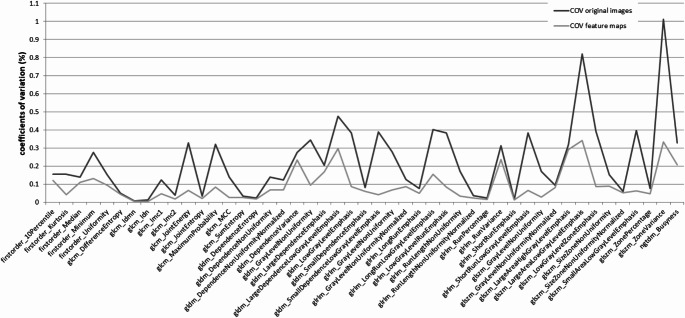
Features with improving reproducibility by parametric map extraction. When features of the 61 included patients were retrieved from the parametric feature maps, coefficients of variation (COVs) of 44 features (of the total extracted 93 features) improved (light gray line) compared to conventional extraction from the original images (dark gray line)

When derived from the original images, 24 out of 93 features showed COVs < 10%. When extracted from the parametric feature maps, COVs for 44 features notably improved, as shown in Fig. 6, of which 39 features revealed COVs < 10%. COVs across all 61 included patients are provided in the supplementary material S4 (extraction from original images) and S5 (extraction from parametric feature maps).

### Experimental setting

**Fig. 7:**
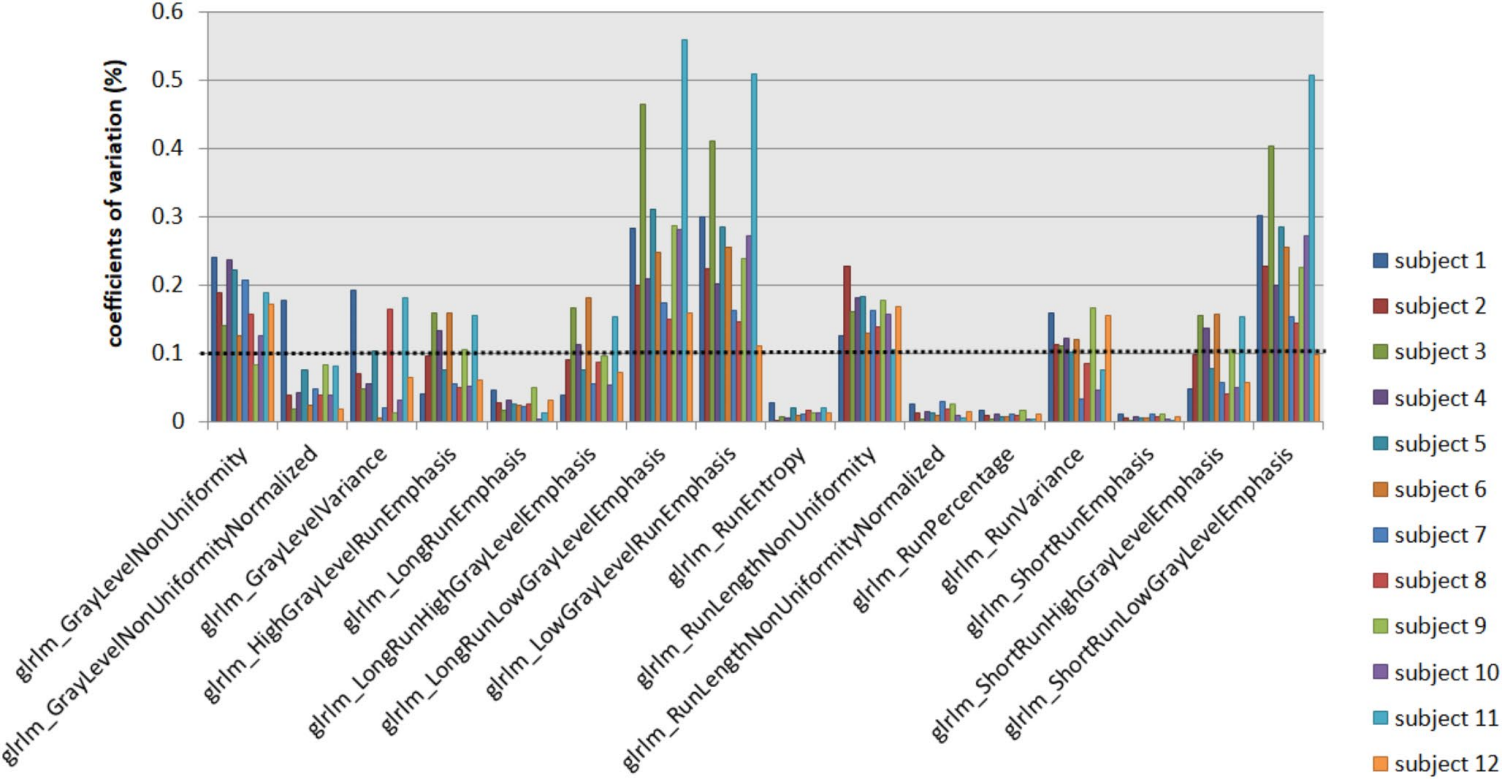
Coefficients of variation of the GLRLM feature group derived from the original images. Coefficients of variation (COVs) of the GLRLM features for the 12 study subjects are plotted. The cutoff at 0.1 (dotted line) indicates reproducibility, with most features showing COVs > 0.1

**Fig. 8:**
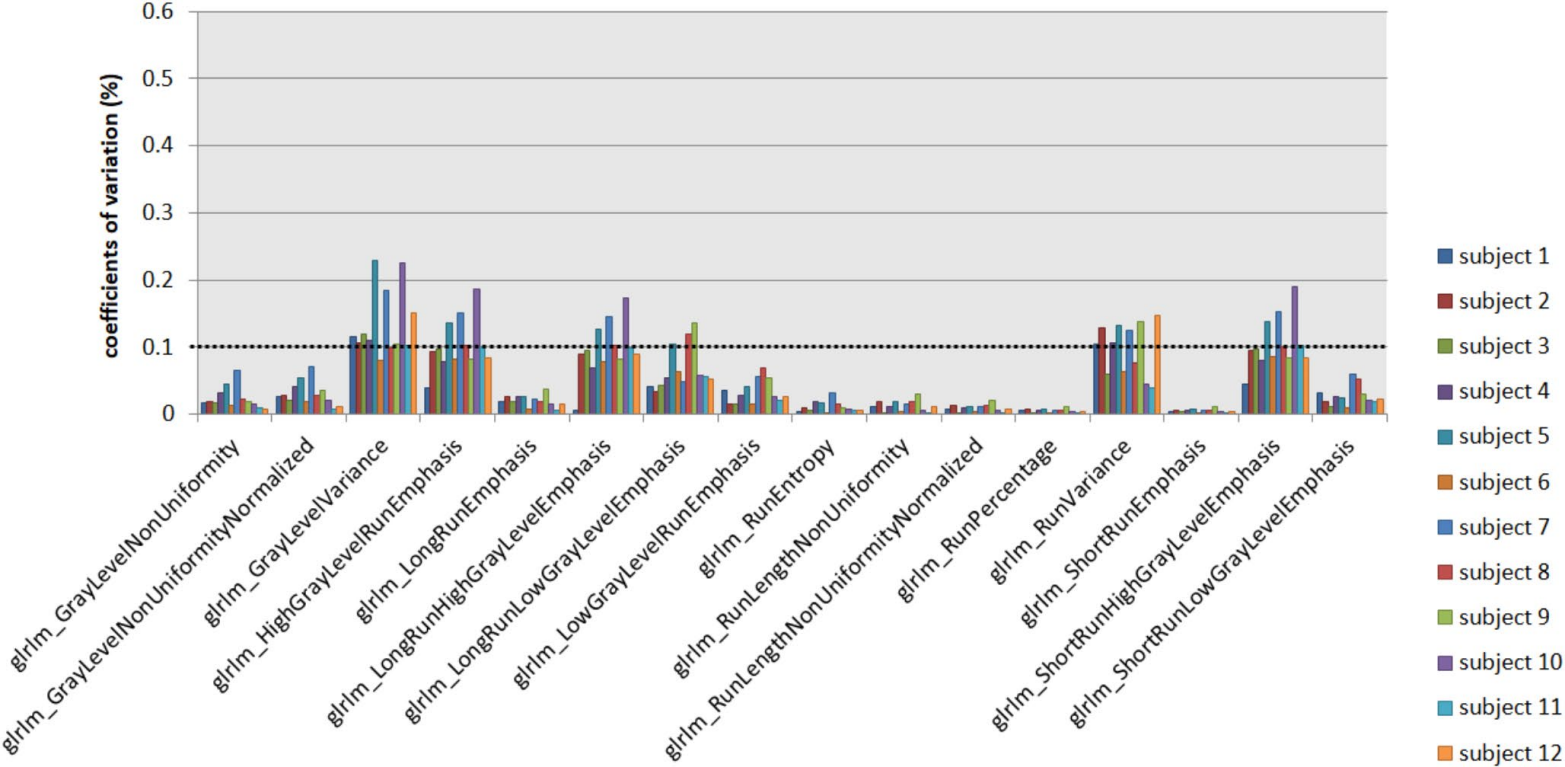
Coefficients of variation of the GLRLM feature group derived from the parametric maps. Coefficients of variation (COVs) of the GLRLM feature group are shown for the 12 study subjects. Most of the features show COVs < 0.1 (dotted line), indicating reproducibility

**Fig. 9:**
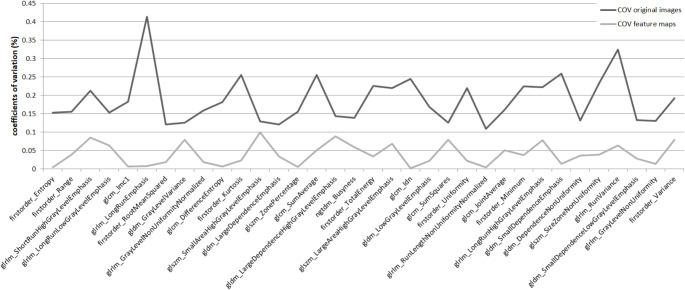
Features with increasing reproducibility by parametric map extraction. Exemplary data from one study subject is shown. The two lines represent features with coefficients of variation changing from > 0.1 in the extraction from the original images (dark gray) to < 0.1 following parametric map extraction (light gray)

When derived from the original images, 29 out of 93 extracted features revealed COVs < 10% across all FOVs and subjects. When features were derived from the parametric maps, 48 features showed COVs < 10%. Of the total of 1116 calculated COVs (93 COVs for 12 subjects), 693 (62%) were < 10% when features were extracted conventionally and 898 (80%) following parametric map extraction. Figure 7 overviews, as an example, the GLRLM feature group derived from the original images and Fig. 8 from the parametric maps across the different FOVs. Figure 9 shows the COVs of the first study subject that changed from > 10% to < 10% following the extraction from the parametric feature maps compared to extraction from the original images. Detailed numerical results on the COVs are provided in the supplementary material (COVs of the extraction from the original images in S6 and the parametric feature maps in S7).

#### Summary of results

In the experimental setting, the number of reproducible features across different FOVs raised from 29 to 48 (of a total of 93 extracted features) by 66% when derived from the parametric maps instead of conventional feature extraction from the original images. The number of reproducible features increased from 24 to 39 in the clinical setting (63%), with the reproducible features overlapping with the experimental study part.

## Discussion

This study shows that parametric feature maps improve the reproducibility of radiomics across different FOVs in cardiac cine images. In the parametric feature map approach, the original image is dissembled into voxels of a fixed size (adjustable in the software script), and the quantity of the feature is calculated for each voxel separately in contrast to the conventional feature extraction, where the feature is calculated from a ROI or VOI from the original image. Since FOVs in cardiac cine images are adjusted individually for each patient and, therefore, are arbitrary, parametric maps can be beneficial in assessing feature quantity from cardiac cine images once radiomics are translated into clinical care. For example, computing parametric maps could be useful when extracting delta radiomics of patients examined with different FOVs over time.

Beyond improving reproducibility across different FOV sizes, another benefit of the parametric maps is that the influence of varying ROI sizes on radiomics, i.e., volume confounding, is mitigated, which was shown in CT images of a water phantom and liver MR images [22, 23]. These effects may also have influenced the results of the presented study. Differently sized ROIs, due to different left ventricular wall thickness, for example, are likely to have been partially compensated for by the maps. Although there are overlaps in reproducible features between the presented study and the analyses of VOI dependencies in previous studies, particularly for second-order features (e.g., the GLRLM features), the compensation of different VOI sizes included more reproducible features overall. Other positive aspects of the parametric feature map approach are that the impact of single-pixel outliers (e.g., caused by artifacts) on the feature quantity is reduced and that feature quantity can be visually displayed for the entire input image, elucidating the individual feature’s behavior [23, 24]. The PyRadiomics settings used for map calculation are deliberately basic. For instance, no filters were applied. While we recognize that numerous other options are available that might enhance or potentially worsen the results, our choice was influenced by the need to balance computational efficiency with practicality. Nevertheless, testing alternative settings holds promise for further improving the feature map approach.

Myocardial tissue characterization based on radiomics from cine images could allow objective image analysis or enhance diagnosis if LGE is unclear or unavailable [4, 34]. As known, radiomics are prone to different acquisition parameters (e.g., slice thickness, flip angle, repetition time) and scanner manufacturers [21, 35, 36]. Although there are recommended imaging parameters for CMR, modifications concerning the patients’ anatomy, heart rate, and specific absorption rate are unavoidable [21]. Thus, standardizing imaging parameters, as suggested for other imaging modalities, is not an option [13]. Knowledge about the susceptibility of CMR-derived radiomics and techniques to improve their robustness is a crucial step in translating radiomics into clinical image analysis [21]. A multi-center and multi-vendor study by Raisi-Estabragh et al. showed wide variability in the repeatability of radiomics derived from short-axis cine bSFFP in each feature class [29]. In a test-retest study on 10 healthy participants and 51 patients analyzing cine bSFFP, T1- and T2-weighted images, and T1/T2 relaxometry images, Jang et al. investigated the reproducibility of myocardial radiomics. They found the GLRLM feature group the most robust in cine bSFFP [26]. In our study, the GLRLM features were pivotally affected by different FOV sizes, and their reproducibility significantly improved when derived from the parametric feature maps. Hence, parametric feature maps could be implemented in study designs to enhance the generalizability of the results. For example, Schofield et al. attempted to differentiate etiologies of LV hypertrophy (hypertrophic cardiomyopathy, cardiac amyloid, aortic stenosis, hypertension) with textural features derived from cine images, including the first-order feature kurtosis [34]. In the presented study, the reproducibility of kurtosis was poor when extracted from the original images and significantly improved by parametric map extraction.

Baessler et al. also presented parametric maps of higher-order texture features in a study on cardiac MRI intending to separate scar tissue from healthy myocardium [37]. While their maps were used to visually assess the quantity, our parametric map technique also allows direct retrieval of the numeric feature quantity by placing a ROI and extracting the mean, comparable to an ADC map in clinical imaging. Therefore, a quick assessment of the feature quantity of any area in the image (e.g., a myocardial segment or a focal lesion in partially included liver parenchyma) is possible. Once radiomics are introduced in clinical care, parametric maps could facilitate clinical decision-making and serve as an objective and quantitative imaging tool, conceivably supporting precision diagnoses in personalized medicine [38]. For example, applying parametric feature maps in clinical imaging might facilitate the visual analysis of cardiac stress perfusion MRI and reduce inter-observer variability [39, 40].

Our study has some limitations. The discrepancy between the number of included patients and the number of healthy individuals might serve as a source of bias and limit the generalizability of our findings. Further, only healthy individuals and patients with normal cardiac MRI and absent cardiovascular pathologies in the medical record were included. None of the included individuals underwent cardiac biopsy, and LGE images were not acquired for the healthy volunteers. Therefore, the absence of myocardial pathologies must be assumed but cannot be entirely excluded. Furthermore, radiomics were extracted from a two-dimensional ROI instead of segmenting the entire LV myocardium three-dimensionally, which would have allowed a more comprehensive capture of tissue properties. Also, the patients in the clinical setting were examined in the clinical routine on the same scanner using a standardized imaging protocol, which includes the individual adjustment of the left-ventricular angulation in the short axis for the individual patient, possibly influencing radiomics beyond the different FOVs. Furthermore, different heart rates of the patients and healthy individuals may have affected image quality and, therefore, radiomics of the cine images [41, 42]. Yet, the applied temporal resolution of 35–44 ms is considered adequate in most patients [43, 44]. Nonetheless, the reproducibility of radiomics from the images from clinical care and the experimental setting was enhanced by computing parametric feature maps. Thus, the parametric maps present a possible step in translating radiomics into clinical application. Yet, the reproducibility across different FOVs was only tested in short-axis cine images and only in a collective without myocardial pathologies, and therefore, the generalizability of our findings must be questioned and assessed further.

In conclusion, parametric feature maps improve the reproducibility of radiomics across different FOVs in cardiac cine images. They can contribute to the reproducibility of radiomics in clinical trials or, perspectively, clinical applications. As a next step, a study on patients with myocardial pathologies examined prospectively with different FOVs could enhance the generalizability of the presented findings.

## Electronic supplementary material

Below is the link to the electronic supplementary material.

Supplementary Material 1

Supplementary Material 2

Supplementary Material 3

Supplementary Material 4

Supplementary Material 5

Supplementary Material 6

Supplementary Material 7

## Data Availability

No datasets were generated or analysed during the current study.
