# Supplementary material for: Effects of parametric feature maps on the reproducibility of radiomics from different fields of view in cardiac magnetic resonance cine images– a clinical and experimental study setting
Source: Int J Cardiovasc Imaging. 2025 Apr 23;41(6):1173–84. doi: 10.1007/s10554-025-03404-y (PMC12162737; doi:10.1007/s10554-025-03404-y)
Supplement: Supplementary file 1 — Supplementary Material 1 [file 10554_2025_3404_MOESM1_ESM.docx]

MRI Indications (corresponding number of patients in parentheses)

Tachycardia (5)

Fatigue (2)

Chest pain (14)

Hypesthesia of the left arm (1)

Unclear findings from the outpatients (3)

Bigeminus (1)

Syncope (5)

Dyspnea (3)

Post-infection (including Covid19, influenza, infectious mononucleosis) to rule out myocarditis (10)

Exclusion of myocarditis after vaccination (1)

Abnormalities in the ergometry (1)

T-negativations in the ECG (1)

ECG changes in general (4)

Suspicion of ARVC (3)

Ventricular extrasystole (5)

Palpitations (2)

Comorbidities

Depression (without medication) (1)

Depression (with medication) (2)

Post diverticulum surgery of the urinary bladder in childhood (1)

Post-surgical treatment of pyloric stenosis in childhood (1)

Bronchial asthma (without medication) (1)

Bronchial asthma (with medication) (2)

Crohn’s disease and PSC (1)

Diabetes mellitus type 1 (insulin therapy) (1)

Post appendectomy over 10 years ago (2)

Post inguinal hernia surgery (1)

Post-cholecystectomy (1)

Post-splenectomy (1)

Epilepsy (3)

PFO (1)

Optic neuritis (1)

Hypothyroidism (2)

Migraine (1)

axSpa (1)

Grave’s disease (1)

Medication

Insulin (by injection) (1)

Pantoprazole (4)

Ustekinumab and ursodeoxycholic acid (1)

Levetiracetam, medication paused (1)

Pregabalin, medication paused (1)

Gabapentin (1)

Oxcarbacepine (1)

Dabigatran, Phenprocoumon, and Fluoxetin (1)

Escitalopram (1)

Desloratadine (1)

Fexofenadine (1)

Metoprolol (2)

Carvedilol (1)

Nebivolol (1)

Ramipril, medication paused (1)

Cortisone (1)

Levothyroxine (2)

Thiamazole (1)

Atorvastatin (1)

Salbutamol (1)
