# Supplementary material for: Effects of parametric feature maps on the reproducibility of radiomics from different fields of view in cardiac magnetic resonance cine images– a clinical and experimental study setting
Source: Int J Cardiovasc Imaging. 2025 Apr 23;41(6):1173–84. doi: 10.1007/s10554-025-03404-y (PMC12162737; doi:10.1007/s10554-025-03404-y)
Supplement: Supplementary file 2 — Supplementary Material 2 [file 10554_2025_3404_MOESM2_ESM.docx]

“Effects of parametric feature maps on the reproducibility of radiomics from different fields of view in cardiac magnetic resonance cine images – a clinical and experimental study setting” in the Journal of Cardiovascular Imaging by Jensen L. J., Kim D., Elgeti T., Steffen I. G., Schaafs L.-A., Hamm B., Nagel S.N.; corresponding author: Laura J. Jensen, Charité – Universitätsmedizin Berlin, corporate member of Freie Universität Berlin, Humboldt-Universität zu Berlin, and Berlin Institute of Health, Department of Radiology, Hindenburgdamm 30, 12203 Berlin, Germany; Email: laura-jacqueline.jensen@charite.de

**PyRadiomics Settings**

imageType:

Original: {}

featureClass:

firstorder:

glcm:

glrlm:

glszm:

gldm:

ngtdm:

setting:

binWidth: 5

voxelArrayShift: 300

correctMask: true
